# A High-Fat/High-Sugar Diet Is Associated with Reduced Motor Unit Number and Neuromuscular Dysfunction in Late-Middle-Aged Female Rats: A Pilot Study

**DOI:** 10.3390/life16060946

**Published:** 2026-06-03

**Authors:** Carlos J. Padilla, Samuel R. Hodge, Wiliam Carvajal, Fernando Ferreyro-Bravo, Masatoshi Suzuki, Karla Esbona, Alvaro N. Gurovich, Brian C. Clark, Jeff S. Volek

**Affiliations:** 1 Department of Kinesiology, University of Wisconsin–Madison, Madison, WI 53706, USA; 2 Institute on Aging, University of Wisconsin–Madison, Madison, WI 53706, USA; 3 Department of Human Sciences, The Ohio State University, Columbus, OH 43210, USA; hodge.14@osu.edu (S.R.H.); volek.1@osu.edu (J.S.V.); 4 Subdirectorate of Teaching and Research, Institute of Sports Medicine (IMD), Havana 10800, Cuba; wiliamcarvajal790@gmail.com; 5 Department of Health Sciences, Anáhuac Puebla University, Puebla 72810, Mexico; fernando.ferreyro@anahuac.mx; 6 Department of Comparative Biosciences, University of Wisconsin–Madison, Madison, WI 53706, USA; masatoshi.suzuki@wisc.edu; 7 Department of Pathology and Laboratory Medicine, University of Wisconsin–Madison, Madison, WI 53706, USA; kesbona@wisc.edu; 8 Department of Physical Therapy & Movement Sciences, The University of Texas at El Paso, El Paso, TX 79968, USA; agurovich@utep.edu; 9 Ohio Musculoskeletal and Neurological Institute, Ohio University, Athens, OH 45701, USA; clarkb2@ohio.edu; 10 Department of Biomedical Sciences, Ohio University, Athens, OH 45701, USA; 11 Division of Geriatric Medicine, Ohio University, Athens, OH 45701, USA

**Keywords:** high-fat/high-sugar diet, body composition, neuromuscular function, grip strength, muscle contractility torque

## Abstract

**Background**: Aging is characterized by metabolic dysfunction and neuromuscular decline, and obesogenic diets may exacerbate these processes. High-fat, high-sugar diets (HFHSD) promote adiposity, systemic metabolic dysregulation, and skeletal muscle impairments, yet their impact on motor unit integrity and neuromuscular vulnerability during aging remains unclear. **Methods**: In a controlled preclinical experiment, late-middle-aged (15-mo-old) female F344 rats were randomized to HFHSD (*n* = 6) or regular chow (*n* = 6) for 10 weeks. Longitudinal assessments were conducted at baseline, 6 weeks, and 10 weeks and included body composition, motor unit number estimation (MUNE), forelimb and hindlimb grip strength, gastrocnemius tetanic contractile torque, and post-intervention electrical impedance myography (EIM). Data were analyzed using a two-way mixed-effects ANOVA to assess the effects of diet and time, with statistical significance set at *p* < 0.05. **Results**: HFHSD led to significant increases in body mass and adiposity measures (e.g., abdominal circumference, skinfold thickness). Compared with controls, HFHSD rats exhibited significant reductions in hindlimb MUNE (diet effect, *p* = 0.007) and decreased tetanic contractile torque in both absolute and body mass-normalized values (*p* ≤ 0.002). Absolute forelimb grip strength increased over time (*p* = 0.027), though this effect did not persist after normalization to body mass, and hindlimb grip strength did not differ between groups. EIM at 50 kHz revealed elevated resistance in HFHSD rats (*p* = 0.0497), whereas reactance and phase angle did not differ significantly. **Conclusions**: This pilot study provides preliminary evidence that an HFHSD, initiated during late middle age, may accelerate neuromuscular decline in female F344 rats prior to the typical onset of age-associated motor unit loss. A 10-week HFHSD intervention was associated with reductions in estimated motor unit numbers, impairments in muscle contractility, and a dissociation between absolute and normalized forelimb grip strength outcomes, indicating a potential early vulnerability of the neuromuscular system to obesogenic dietary exposure. These findings should be interpreted within the context of a modest sample size but collectively support the concept that diet-induced metabolic dysfunction may contribute to early neuromuscular impairment during aging.

## 1. Introduction

Late middle age and older adulthood are accompanied by physiological changes that contribute to declines in metabolic and neuromuscular health. Dietary patterns rich in saturated fats, refined sugars, and processed foods are highly prevalent in these populations (and the general population at large) and have been strongly linked to obesity and related metabolic disorders in both humans and laboratory rodents, including visceral fat accumulation, insulin resistance, type 2 diabetes, hepatic steatosis, and hyperglycemia [1,2]. Chronic consumption of high-fat/high-sugar diet (HFHSD) leads to an imbalance between energy intake and expenditure, promoting lipid deposition not only in adipose depots but also in non-adipose tissues such as skeletal muscle, where excess intramyocellular lipid content can impair contractile performance [3,4]. Obesity-related increases in body fat can adversely affect muscle metabolism and contractile function [5,6], accelerating physical decline during aging. Aging itself is characterized by reductions in skeletal muscle mass and function, which contribute to decreased mobility, loss of independence, and elevated risk of falls [7]. Functional impairments during aging are strongly associated with diminished muscle function and the reduced ability of muscle fibers to shorten rapidly under submaximal loads, capacities that are essential for responding to unexpected perturbations during daily activities [8,9,10]. As muscle weakness progresses, older adults experience increasing difficulty performing basic tasks such as rising from a chair, ambulating, and maintaining balance, ultimately compromising autonomy and increasing disability risk.

The basic functional unit governing force production is the motor unit, composed of a motor neuron and the muscle fibers it innervates. Motor unit number estimation (MUNE) provides a sensitive index of motor neuron integrity and early neuromuscular decline [11]. Aging is accompanied by a progressive reduction in the number of functional motor units due to denervation of primarily fast-twitch type II fibers, followed by incomplete or failed reinnervation [12,13]. These neural and muscular alterations impair muscle strength and neuromuscular control, further contributing to mobility loss and decreased functional independence [14]. Although obesogenic high-fat/high-sugar dietary interventions are known to impair metabolism and muscle function, their direct impact on aging motor units is unclear, despite promoting inflammation, mitochondrial stress, and intramuscular fat that may disrupt neuromuscular integrity [15,16]. Given that aging already reduces motor neuron reserve and contractile capacity [17,18], individuals in late middle age may be particularly susceptible to neuromuscular impairments induced by HFHSD. However, it remains unclear whether such diets are sufficient to produce measurable reductions in motor unit function prior to advanced aging. Despite extensive evidence linking HFHSD to metabolic dysfunction, its direct impact on motor unit integrity in late middle age remains largely unexplored.

Middle-aged rodents also exhibit adipocyte adaptations that favor hypertrophy and increased intra-abdominal lipid storage, which may temporarily protect against ectopic lipid spillover into other tissues, including skeletal muscle, but may also predispose animals to later metabolic dysfunction [19]. Considering the established associations between visceral fat, subcutaneous fat, and reduced muscle function, HFHSD consumption has the potential to exacerbate age-related neuromuscular vulnerability; however, this has not been directly examined in late-middle-aged animals.

In this study, we investigated the effects of a 10-week HFHSD in late-middle-aged (15-month-old) F344 female rats, focusing on body adiposity, MUNE, grip strength, and gastrocnemius muscle contractility. Previous work has shown that age-related losses in motor units begin at approximately 18–20 months in mice [20], as well as in F344 rats at 24 months of age [21], raising the question of whether a HFHSD initiated before this period exacerbates this loss. Understanding how obesogenic dietary interventions influence neuromuscular aging is critical given the widespread consumption of these diets among middle-aged and older adults and the growing burden of obesity-related functional decline.

## 2. Methods

### 2.1. Overview and Study Design

**Figure 1:**
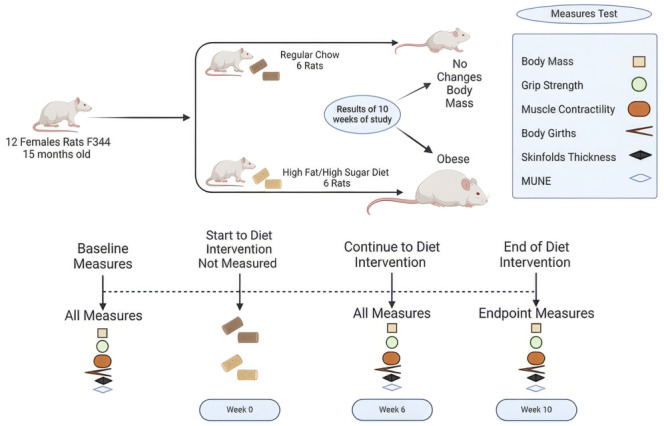
Study design and timeline. This experimental study involved 12 female F344 rats, aged 15 months, which were randomized into two groups: Group 1 (*n* = 6): High-Fat/High-Sugar Diet (Teklad^TM^ Custom Diet—TD.06414; composition: 18.3% protein, 21.4% carbohydrates, 60.3% fat, 12.1% sucrose); Group 2 (*n* = 6): Regular Chow (Teklad—7012; composition: 25% protein, 58% carbohydrates, and 17% fat). The rats were fed *ad libitum* for a duration of 10 weeks. All measurements, including body mass, grip strength, tetanic muscle contractility, body girths, skinfold thickness, and motor unit number estimation (MUNE), were conducted at three time points: baseline, 6 weeks, and 10 weeks.

The longitudinal experimental design of the study is illustrated in Figure 1. Fifteen-month-old female Fischer 344 (F344) rats were obtained from the National Institute on Aging (NIA) Aged Rodent Colony (Charles River Laboratories, Wilmington, MA, USA). This age was selected based on prior studies demonstrating that metabolic adaptations persist into later life stages [22], underscoring the importance of examining middle-aged animals to evaluate long-term physiological consequences. At baseline, rats were evaluated and randomly assigned to either a high-fat/high-sugar diet group (HFHSD; *n* = 6 females, Teklad™ Custom Diet, Inotiv Company, Madison, WI, USA; TD.06414; 18.3% protein, 21.4% carbohydrates, 60.3% fat, with added sucrose [12.1% by weight]) or a regular chow control group (*n* = 6 females, Teklad LM-485 Sterilizable Mouse/Rat Diet, Inotiv Company—7012; 25% protein, 58% carbohydrates, 17% fat), with food provided ad libitum. All animals were obtained from the same vendor and age cohort to minimize source-related variability. Rats were housed three per cage under identical environmental conditions, and individual animals were considered the experimental unit for longitudinal analyses. Although rats were group-housed, weekly cage-level food consumption was monitored throughout the intervention to estimate overall intake patterns. The HFHSD group showed slightly greater food disappearance during the initial weeks of the intervention, with relatively stable intake thereafter. Because the HFHSD had a substantially higher caloric density (5.1 kcal/g) than regular chow (3.1 kcal/g), comparable gram consumption may still have resulted in greater caloric intake. Therefore, the present study cannot fully distinguish whether the observed neuromuscular alterations were driven by diet composition, excess caloric intake, obesity-related metabolic dysfunction, or a combination of these factors. Prior to initiation of the HFHSD intervention, all rats were maintained on the same regular chow diet. The TD.06414 HFHSD was selected for this study because it markedly increases dietary fat content and simple sugar exposure relative to standard chow, thereby mimicking key features of an obesogenic “Western-style” diet that promotes adiposity, insulin resistance, and neuromuscular impairments in rodent models [23]. In contrast, Teklad 7012 is a grain-based maintenance diet with lower fat content, higher complex carbohydrates, and no added sucrose, and is not typically associated with spontaneous obesity or metabolic dysfunction [24]. Using this well-characterized HFHSD versus a standard chow control allows us to model diet-induced obesity and metabolic stress under controlled conditions, isolate the impact of chronic high fat and sugar intake on neuromuscular outcomes, and enhance the translational relevance of our findings to human diets that are rich in saturated fat and refined sugars.

This investigation was intentionally designed as a pilot study to establish feasibility, generate preliminary effect-size estimates, and identify neuromuscular outcomes most sensitive to diet-related changes. The repeated-measures longitudinal design increases the precision of within-animal comparisons over time, reducing variability and allowing meaningful interpretation even with small cohorts. A sample size of six rats per group is consistent with prior pilot and mechanistic neuromuscular aging studies and is appropriate for detecting biologically relevant trends, characterizing variability, and informing power calculations for subsequent definitive trials. Rather than aiming to test all hypotheses with full statistical power, this pilot study focuses on identifying clear directional effects, refining protocols, and supporting the design of larger, confirmatory experiments [25,26,27]. Accordingly, the present findings should be interpreted as preliminary and associative rather than definitive evidence of causal neuromuscular mechanisms.

Female rats were selected because aging-related hormonal fluctuations increase susceptibility to dysregulated energy metabolism and adiposity-related body composition changes [28,29,30,31,32]. Longitudinal assessments were conducted at baseline (0 weeks), 6 weeks, and 10 weeks, which included body mass, body circumferences, skinfold thickness, motor unit number estimation, grip strength, and tetanic muscle contractility. All outcome assessments were performed by personnel blinded to group assignment. All procedures were approved and conducted in accordance with the Institutional Animal Care and Use Committee at the University of Wisconsin–Madison (Protocol M006781; approval date: 1/11/2024).

### 2.2. Rats’ Diet Composition in the Study

The F344 rats used in this study were divided into two groups, each receiving one of two different diets: a high-fat/high-sugar diet (TD.06414) or regular chow (7012, Teklad LM-485 Sterilizable Mouse/Rat Diet). Both diets were formulated by Teklad™ Custom Diets (Madison, WI, USA). The high-fat/high-sugar diet (TD.06414-Purified Diet or Refined Ingredients Diet) contains approximately 60% of total calories derived from fat [33]. The fatty acid profile based on total fat content is as follows: 36% saturated fat, 41% monounsaturated fat, and 23% polyunsaturated fat. The formulation of the HFHSD includes the following ingredients (in g/kg): Casein, L-Cystine, Maltodextrin, Sucrose, Lard, Soybean Oil, Cellulose, Mineral Mix, Ain-93G-MX (94046), Calcium Phosphate, Dibasic, Vitamin Mix, AIN-93-VX (94047), Choline Bitartrate, and Blue Food. The regular chow diet (7012) is a fixed formula grain-based diet designed to be sterilized in an autoclave to support rodent growth [33]. Its ingredient composition (in descending order) includes: Ground corn, dehulled soybean meal, ground oats, wheat middling’s, dehydrated alfalfa meal, soybean oil, corn gluten meal, calcium carbonate, dicalcium phosphate, brewers dried yeast, iodized salt, choline chloride, magnesium oxide, L-lysine, DL-methionine, ferrous sulfate, menadione sodium bisulfite complex (source of vitamin K activity), vitamin E acetate, thiamin mononitrate, calcium pantothenate, manganous oxide, niacin, copper sulfate, zinc oxide, vitamin A acetate, pyridoxine hydrochloride, riboflavin, vitamin D3 supplement, vitamin B12 supplement, folic acid, biotin, calcium iodate, and cobalt carbonate. Additionally, diet 7012 was supplemented with extra vitamins to ensure nutritional adequacy following autoclave treatment.

It is important to note that the high-fat/high-sugar diets used in rodent studies can vary in macronutrient distribution, ranging from values of 45% fat [34] to approximately 60% [35]. For this study, we used a diet with 60.3% fat as a proportion of the total energy consumed. There are also diets with even higher fat content, such as the ketogenic diet, which contributes 90% of energy from lipids [35]. The ketogenic diet promotes different adaptive responses at both physiological and musculoskeletal levels in the rats that consume it [24].

### 2.3. Anesthesia and Preparation Protocols

During body composition measurements, electrophysiological recordings, and tetanic muscle contractility assessments, inhaled isoflurane was administered for anesthesia. The induction phase utilized a concentration of 3–5%, followed by maintenance at 2–3%, using a Somnosuite^®^ low-flow anesthesia system (Kent Scientific, Torrington, CT, USA). To ensure the well-being of the rats, body temperature was maintained at 37 °C with the aid of an infrared heating pad (Kent Scientific). To prevent corneal dryness and irritation during anesthesia, a petroleum-based eye lubricant (Dechra, Northwich, UK) was applied. Prior to the procedures, hair was removed from the right hindlimb using a Remington model VPG 6530 (DeForest, WI, USA) to facilitate accurate electrode placement for electrophysiological recordings. Notably, rats were not anesthetized during the muscle grip strength test to ensure the accuracy of the measurements in a conscious state.

### 2.4. Body Composition (Body Mass, Girths, and Skinfolds Thickness)

**Figure 2:**
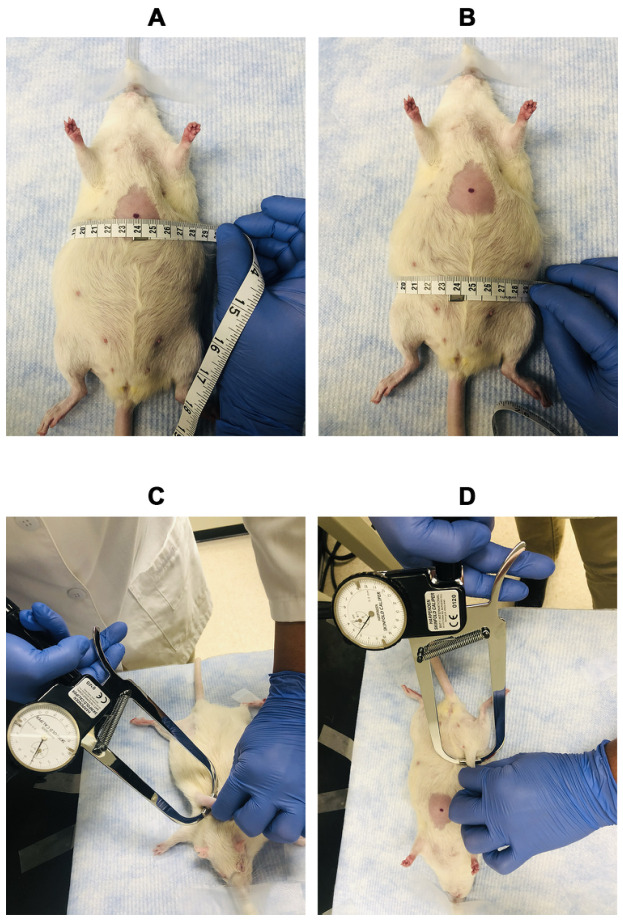
Girth and skinfold measurements in rats. Rats were placed in the supine position and stabilized. Girths were measured at (**A**) the chest (xiphoid level) and (**B**) the abdomen (~2.5 cm below the umbilicus). Skinfold thickness was measured with a Harpenden caliper at the scapular (**C**) and abdominal (**D**) sites.

All F344 rats were weighed weekly (in grams) throughout the 10-week intervention. Body circumferences were measured using a Lufkin cloth tape marked in centimeters. For girth measurements, rats were placed in the supine position and gently stabilized. While in this position, the tape was stretched (1) around the chest at the level of the xiphoid and (2) around the abdomen approximately 2.5 cm below the umbilical level [36,37]. While the rat was still stabilized, skinfold measurements (scapular and abdominal) were performed with a Harpenden skinfold caliper (Figure 2). Readings were taken in millimeters while holding the caliper horizontally relative to the upright posture of the rat. These two skinfolds were selected as they have previously provided valuable information on fatness in both humans and rats [36,37]. The measurements helped to quantify fat mass and overall changes in adiposity in response to the HFHSD compared to regular chow.

### 2.5. Assessment of Grip Strength

Bilateral forelimb and hindlimb grip strength were evaluated using a force transducer (Model BIO-GS4, Bioseb SAS, BP32025-F-13845, Vitrolles Cedex, Pinellas Park, FL, USA) [14,38]. Rats were securely grasped to ensure stability during the assessment. When a rat grasped the transducer bar with its forelimbs or hindlimbs, a steady, constant pull was applied until it released its grip. Each rat underwent three trials for both forelimb and hindlimb grip-strength assessments. The average value from the three trials for each limb was calculated and used for subsequent analyses.

### 2.6. Muscle Contractility Torque Measurement

To evaluate muscle contractility, each anesthetized rat was positioned in a supine position, with the right hindlimb paw secured to a force plate using Transpore medical tape (3M, Maplewood, MN, USA). The distal femoral condyles of the knee were secured to the platform using blunt clamps to ensure stability during measurements, and the paw and foot were aligned with the tibia at a 90° angle with the knee kept extended. A pair of disposable monopolar electrodes (Natus Neurology Inc., Middleton, WI, USA) was inserted subcutaneously into the medial posterior region of the leg, near the tibial branch of the sciatic nerve. To assess maximal twitch torque, individual 0.20 ms square wave pulses were delivered, and stimulus intensity gradually increased until the maximum twitch response was achieved. Following this, maximal tetanic torque was evaluated using supramaximal stimuli (120–150% of maximum intensity) delivered at a frequency of 150 Hz in a 900 ms train to induce tetanic contraction. This stimulation frequency and train duration (150 Hz, 900 ms) were selected based on a prior force–frequency evaluation conducted in these rats [14]. For comparative analysis, both absolute and body mass–normalized maximum torque values were recorded. All in vivo contractility assessments were performed using the 1301A Isometric Whole Animal System (Aurora Scientific Inc., Aurora, ON, Canada) for mice, a platform specifically designed for isometric footplate experiments. This system allows for the evaluation of in vivo isometric muscle function, and our methodology aligns with previously published protocols in mouse and rat models [14,24,39,40,41].

### 2.7. Evaluation of Motor Unit Number Estimation

Motor unit number estimation is an electrophysiological technique utilized to assess the functional status of the motor unit pool in vivo [42]. In this study, in vivo electrophysiological measures, including compound muscle action potential (CMAP) amplitude, mean single motor unit potential (SMUP) amplitude, and motor unit number estimation (MUNE), were obtained from the hindlimbs of the rats using an electrodiagnostic system (Sierra Summit Clinic, Cadwell, Kennewick, WA, USA) [14,24]. For hindlimb recordings, the rats were positioned supine, and the right hindlimb was secured to the recording stage with Transpore medical tape (3M). Surface disk electrodes (TECA 6030-TP, Natus Neurology Inc., Middleton, WI, USA) were lightly coated with conductive gel (Parker Labs, Fairfield, NJ, USA) and placed over the skin above the gastrocnemius muscle (electrode E1) and over the calcaneus (electrode E2). Nerve stimulation was conducted using a pair of 28-gauge monopolar needle electrodes, designated as the cathode and anode, which were placed subcutaneously in the region of the sciatic nerve in the proximal thigh. During compound muscle action potential recordings, a supramaximal stimulus (120% of the maximum) was delivered to the right sciatic nerve. The sensitivity of the recording device was set to 200 mV, with a display of 20 mV per division, and the duration was configured for 10 ms, with 1 ms per division. Peak-to-peak compound muscle action potential values were recorded in millivolts (mV). Incremental stimulation was applied to obtain a total of 10 incremental compound muscle action potential responses, which were averaged to determine the mean single motor unit potential size. Motor unit number estimation values for the hindlimbs were calculated using the formula: MUNE = average CMAP ÷ SMUP.

### 2.8. Statistical Analysis

Statistical analyses were performed using GraphPad Prism Version 11.0.0 (GraphPad Software, Inc., San Diego, CA, USA). Body mass and morphometric measures (abdominal girth, chest girth, scapular skinfold, and abdominal skinfold), tetanic muscle contractility (torque), grip strength (forelimb and hindlimb), and electrophysiological data (motor unit number estimation) were tested for normality using the Shapiro–Wilk test and were normally distributed. These variables were analyzed using two-way mixed-effects ANOVA (diet and time × diet interaction). Given the pilot/feasibility nature of this study, eta-squared effect sizes (η^2^) were calculated to estimate the magnitude of the effects of the high-fat/high-sugar diet on body mass and morphometric measures in late-middle-aged female rats and to inform the design of future adequately powered studies. A *p*-value < 0.05 was considered statistically significant. Given the pilot nature and modest sample size of the study, the statistical analyses and longitudinal findings should be interpreted as exploratory rather than definitive.

## 3. Results

The findings of this study show that high-fat feeding during middle-aged adulthood induced significant increases in body mass and central adiposity, accompanied by a marked decline in motor unit number and tetanic torque, reflecting early neuromuscular deterioration (Table 1).

### 3.1. High-Fat/High-Sugar Diet Induces Adiposity-Related Increases in Body Composition Measures

**Figure 3:**
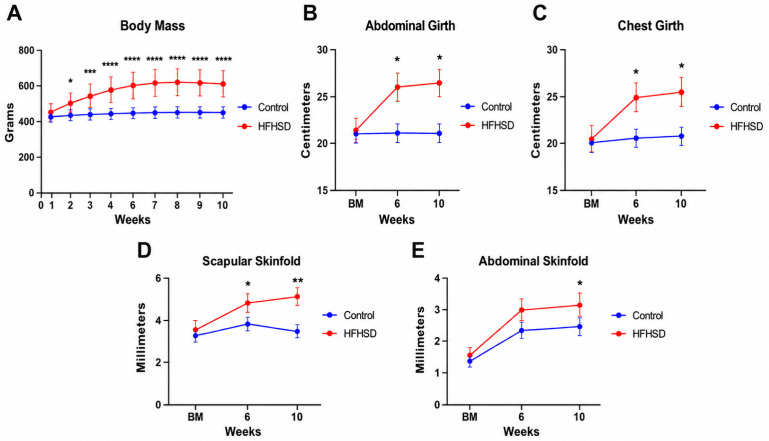
Body composition measures in late-middle-aged F344 rats fed a high-fat/high-sugar diet (HFHSD) or regular chow (Control). (**A**) Body mass showed a significant increase over the 10-week intervention in the HFHSD group (η^2^ = 0.661, two-way mixed-effects ANOVA time × diet, *p* = 0.0046). Significant HFHSD-induced increases were also observed for (**B**) abdominal girth (η^2^ = 0.543, *p* = 0.0001), (**C**) chest girth (η^2^ = 0.444, *p* = 0.0002), (**D**) scapular skinfold (η^2^ = 0.416, *p* = 0.0008), and (**E**) abdominal skinfold (η^2^ = 0.512, *p* = 0.006). Statistical significance symbols placed directly above individual data points indicate significant differences between HFHSD and control groups at the corresponding time point. Šídák’s multiple comparisons test adjusted *p*-values: * *p* < 0.05, ** *p* < 0.01, *** *p* < 0.001, **** *p* < 0.0001. BM = baseline measurements obtained prior to the dietary intervention. Control groups are represented in blue, whereas HFHSD groups are represented in red.

The HFHSD significantly affected body composition by increasing body mass (η^2^ = 0.661) (Figure 3A), abdominal girth (η^2^ = 0.543) (Figure 3B), pectoral girth (η^2^ = 0.444) (Figure 3C), scapular skinfolds (η^2^ = 0.416) (Figure 3D), and abdominal skinfolds (η^2^ = 0.512) (Figure 3E) over the 10-week study period in late-middle-aged female rats. Notably, several of these adiposity-related changes were already evident at 6 weeks and remained relatively stable through week 10.

### 3.2. High-Fat/High-Sugar Diet Reduces Motor Unit Number Estimation in Late Middle-Aged Rats

**Figure 4:**
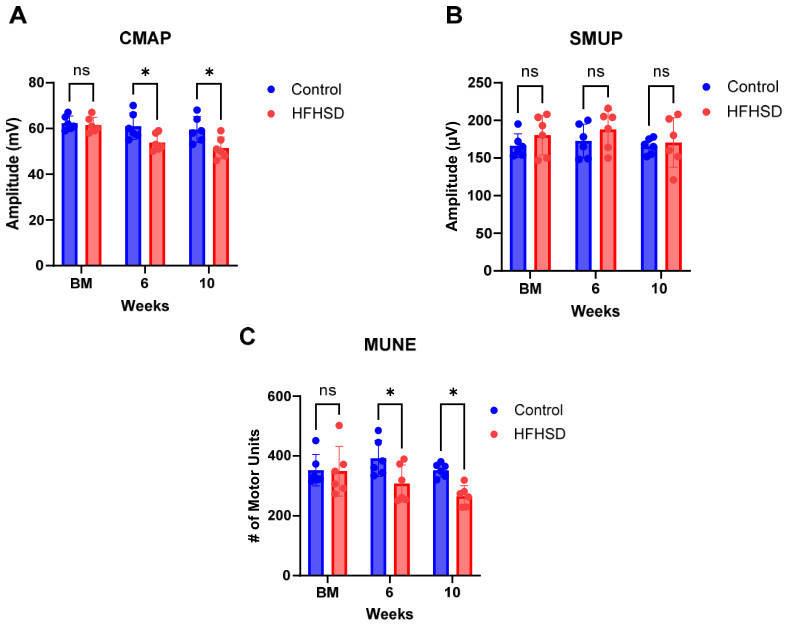
Late-middle-aged female F344 rats fed a high-fat/high-sugar diet (HFHSD) for 10 weeks showed a reduction in compound muscle action potential (CMAP) and in motor unit number estimation (MUNE) in the gastrocnemius muscle during aging. HFHSD rats demonstrated a significant reduction over the 10-week period in (**A**) CMAP (two-way mixed-effects ANOVA, time × diet, *p* = 0.0219) but not in (**B**) single motor unit potential (SMUP) (two-way mixed-effects ANOVA, time × diet, *p* = 0.6898). Significant reduction was also observed in (**C**) MUNE (two-way mixed-effects ANOVA, diet, *p* = 0.0071) compared with the regular chow-fed F344 group. Šídák’s multiple comparisons test adjusted *p*-values: *p* < 0.05. BM = Baseline Measures; mV = millivolts; µV = microvolts. * *p* < 0.05; ns, not significant.

The MUNE is a valuable electrophysiological technique for assessing neuromuscular function during aging, as it provides a direct measure of the number of functional motor neurons or axons innervating a muscle or muscle group [43,44]. In addition to being a very useful technique to analyze neuromuscular function, it is also useful to evaluate the degree of loss of motor units associated with aging, motor neuron disorders, or peripheral neuropathies and can provide data of great value when evaluating the results of treatments for these disorders [45]. Several neuromuscular alterations induced by the HFHSD were already evident at 6 weeks and persisted through week 10. The HFHSD significantly reduced the CMAP (peak-to-peak; *p* = 0.0219) (Figure 4A) as well as MUNE (*p* = 0.0071) in late-middle-aged F344 rats (Figure 4C), but did not affect SMUP (*p* = 0.6898) (Figure 4B).

### 3.3. High-Fat/High-Sugar Diet Impairs Muscle Contractility and Reveals Dissociation Between Absolute and Normalized Strength

**Figure 5:**
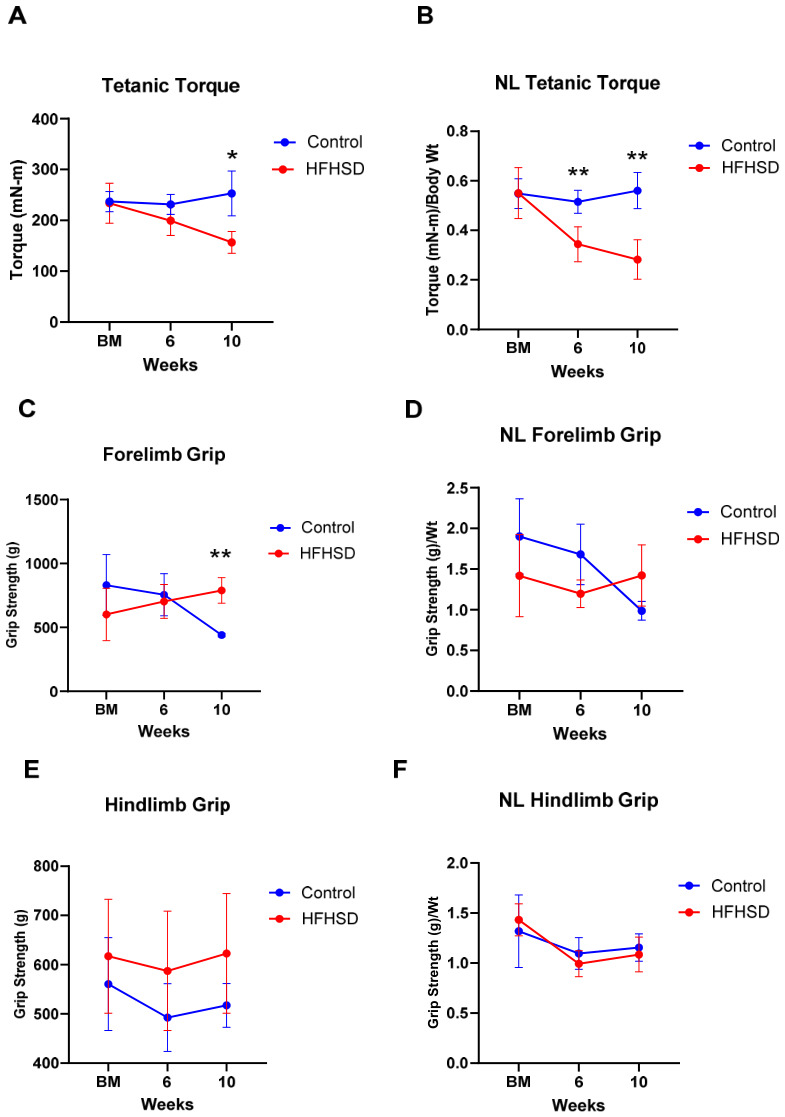
High-fat/high-sugar diet (HFHSD) reduces tetanic muscle contractility for 10 weeks, improves forelimb grip but not normalized (NL) forelimb grip, and does not affect hindlimb grip in aged female rats. In late-middle-aged female rats, an HFHSD led to a significant reduction in (**A**) tetanic torque (two-way mixed-effects ANOVA time × diet, *p* = 0.0023) and (**B**) NL tetanic torque (two-way mixed-effects ANOVA time × diet, *p* = 0.0001). There was a significant increase in (**C**) forelimb grip (two-way mixed-effects ANOVA time × diet, *p* = 0.0273), but not in (**D**) NL forelimb grip (two-way mixed-effects ANOVA time × diet, *p* = 0.1924). Additionally, there were no significant changes in (**E**) hindlimb grip (two-way mixed-effects ANOVA time × diet, *p* = 0.6281) or (**F**) NL hindlimb grip (two-way mixed-effects ANOVA time × diet, *p* = 0.2323). Šídák’s multiple comparisons test adjusted *p*-values: * *p* < 0.05, ** *p* < 0.01. BM = Baseline Measures.

The HFHSD significantly reduced absolute tetanic torque (time × diet, *p* = 0.0023), normalized tetanic torque by body mass (time × diet, *p* = 0.0001), and forelimb grip strength (time × diet, *p* = 0.0273) in F344 rats (Figure 5A–C). Most of these changes were already apparent at 6 weeks and remained relatively stable through week 10. However, the high-fat/high-sugar diet intervention did not significantly affect normalized forelimb grip (time × diet, *p* = 0.1924), absolute hindlimb grip (time × diet, *p* = 0.6281), or normalized hindlimb grip (time × diet, *p* = 0.2323) compared with the regular chow diet (Figure 5D–F). The dissociation between absolute and body mass-normalized outcomes suggests that increases in absolute force may reflect greater body mass and mechanical loading, whereas normalized measures more accurately reflect muscle quality and neuromuscular efficiency [46].

### 3.4. High-Fat/High-Sugar Diet Alters Muscle Electrical Properties at 50 kHz

**Figure 6:**
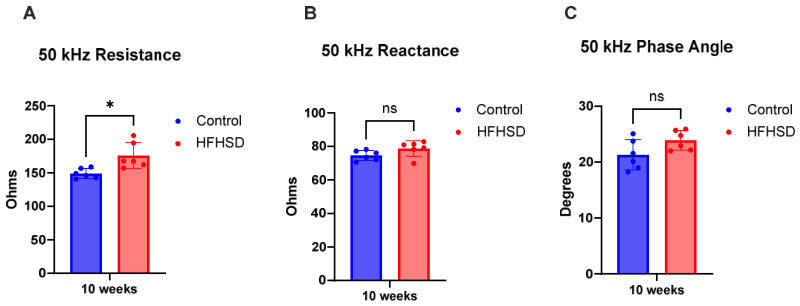
Electrical impedance myography measures at 50 kHz following the HFHSD intervention at 10 weeks. (**A**) Resistance was significantly higher in HFHSD-fed rats compared with controls (unpaired t-test, *p* = 0.0497). (**B**) Reactance showed no significant difference between groups (*p* = 0.0922). (**C**) Phase angle also did not differ significantly between groups (*p* = 0.3150). * *p* < 0.05. ns, not significant (*p* ≥ 0.05).

Electrical impedance myography (EIM) parameters were analyzed at 50 kHz, the most commonly reported frequency in rodent EIM studies [47]. The high-fat/high-sugar diet altered muscle electrical properties at this frequency, with group differences in phase, resistance, and reactance consistent with diet-induced changes in muscle tissue. HFHSD rats showed significantly higher resistance compared with controls (*p* = 0.0497), indicating potential increases in non-contractile tissue or alterations in muscle electrical conduction properties, as increases in resistance have been associated with muscle fiber atrophy and greater non-contractile tissue content in EIM studies [48,49]. In contrast, reactance (*p* = 0.0922) and phase angle (*p* = 0.3150) did not differ significantly between groups, suggesting that membrane capacitance and overall muscle health were not markedly altered after 10 weeks of HFHSD consumption (Figure 6).

## 4. Discussion

In the present study, we investigated whether an obesogenic high-fat/high-sugar diet (HFHSD) affects neuromuscular function, muscle contractility, and strength in late-middle-aged rats. These findings should be interpreted within the context of a pilot study with a modest sample size; therefore, mechanistic conclusions remain preliminary and require confirmation in larger studies. Previous rodent studies have associated HFHSD exposure with obesity, systemic inflammation, insulin resistance, and metabolic dysfunction. However, metabolic parameters such as glucose, insulin, circulating lipids, inflammatory markers, liver fat accumulation, and insulin resistance indices were not directly assessed in this study. Consequently, the observed neuromuscular alterations should be interpreted as changes associated with an obesogenic dietary intervention rather than direct evidence of metabolic dysfunction. In addition, the HFHSD and regular chow diets differed in several nutritional characteristics beyond fat and sugar content, including caloric density, fiber content, ingredient composition, and macronutrient distribution. Therefore, the findings should not be attributed exclusively to fat or sugar alone, but more broadly to the overall obesogenic dietary exposure. For example, He et al. (2020) reported that 21 weeks of a high-fat diet in C57BL/6J young mice produced adipose tissue expansion and insulin resistance, while Kappe et al. (2014) showed that 10 weeks of HFHSD caused significant weight gain, fasting hyperglycemia, and hyperinsulinemia [50,51]. Messa et al. (2020) further demonstrated that HFHSD-induced intramyocellular lipid accumulation appears earlier in older mice compared to younger mice, negatively affecting muscle function [6]. These prior findings provide a physiological rationale for investigating HFHSD-induced neuromuscular decline in late middle age. However, because food intake and caloric intake were not directly quantified at the individual level and pair-feeding was not implemented, the present findings cannot determine the relative contribution of dietary composition versus excess energy intake to the observed neuromuscular alterations.

### 4.1. High-Fat/High-Sugar Diet and Body Composition in Late-Middle-Aged Rats

Anthropometric measurements remain a practical tool for estimating body composition in rodents and humans [51,52]. In the present study, HFHSD-fed rats exhibited significant increases in body mass, chest and abdominal girths, and scapular and abdominal skinfold thickness, consistent with elevated adiposity (Figure 3). Comparable results have been reported by Melo et al. (2021), who observed rapid increases in body weight and adipose-related metabolic disturbances after 30 days of high-fat and fructose supplementation [53]. Similarly, Han et al. (2021) found that one-month-old C57BL/6J mice fed HFHSD for 5 months gained significantly more weight over five months despite comparable caloric intake compared to controls [54]. One likely explanation is that HFHSD increases energy intake due to carbohydrate-driven palatability [55]. Cafeteria-style HFHSD produces even greater hyperphagia and metabolic dysfunction, including hyperinsulinemia, hyperglycemia, hepatic steatosis, and systemic inflammation [56]. The metabolic consequences differ markedly from those of ketogenic diets, even though both are high in fat. Despite similar fat content, HFHSD and ketogenic diets produce fundamentally distinct metabolic states, with opposing effects on insulin signaling, lipid handling, and neuromuscular preservation. Ketogenic diets decrease triglyceride and cholesterol synthesis while upregulating ketogenesis [57,58,59], increase UCP1 expression, and elevate energy expenditure [24,60], enabling weight maintenance in aged rodents [61]. In contrast, HFHSD upregulates lipogenic pathways and increases acetyl-CoA flux into fat storage [57,58], promoting greater adiposity. The present study further suggests that HFHSD increased both subcutaneous and visceral fat, as reflected in girth and skinfold measures. Although intramuscular fat was not directly quantified in our study, the findings align with prior reports of intramyocellular lipid accumulation following HFHSD [6,62]. Chronic adiposity can reduce mobility and muscular function [63], but the present study provides important nuance regarding the role of adiposity in neuromuscular impairment.

### 4.2. High-Fat/High-Sugar Diet Reduces Motor Unit Number Estimation

An important observation of the present study is that several HFHSD-induced alterations were already evident after 6 weeks of dietary exposure and remained relatively stable through week 10. This temporal pattern suggests that obesogenic diets may rapidly induce adiposity-related and neuromuscular disturbances during late middle age, rather than producing only a gradual late-stage decline.

Motor unit number estimation was significantly reduced following HFHSD consumption (Figure 4), consistent with literature showing that diet-induced obesity impairs skeletal muscle and neuromuscular health [5,6,64]. In contrast, ketogenic diets preserve motor units and skeletal muscle function [61,65]. Importantly, the reduction in MUNE observed in HFHSD-fed rats may reflect early functional impairments in motor neuron–muscle connectivity rather than irreversible motor neuron degeneration. Although weekly observations suggested relatively consistent spontaneous cage activity throughout the 10-week intervention, spontaneous physical activity and locomotor behavior were not directly monitored. Therefore, the potential contribution of reduced activity levels to the observed neuromuscular alterations cannot be fully excluded. Moreover, a decline in MUNE also emerged in control rats by week 10 (Figure 4C), likely reflecting age-related neuromuscular remodeling. Since both groups were the same age, the larger reduction in the HFHSD group strongly implicates diet-specific effects rather than mobility or aging alone. Although adiposity can theoretically attenuate surface-detected CMAP and MUNE signals, our laboratory has previously validated surface electrode recordings with excellent reproducibility in F344 rats [38]. In this study, waveform morphology and amplitude were consistent across rats and time points, with identical electrode placement and recording parameters, supporting the technical validity of the MUNE signal and ruling out methodological underestimation. In the present rodent study, EIM parameters were analyzed at 50 kHz, a frequency commonly used in preclinical models [47]. In contrast, human studies have explored multifrequency EIM indices, including phase ratios derived from different frequencies (e.g., 100/300 kHz), to improve discrimination of muscle weakness and alterations in muscle quality [48,49,66]. Although these parameters are not directly comparable across species, both approaches aim to capture alterations in muscle composition and structure. The electrical impedance myography findings further support this interpretation. Resistance at 50 kHz was significantly higher in HFHSD-fed rats, which may reflect alterations in tissue composition associated with HFHSD exposure, including possible increases in extracellular and adipose tissue content. In contrast, reactance and phase angle showed no significant group differences (Figure 6), suggesting relative preservation of muscle membrane integrity and cellular properties. However, because EIM measurements were obtained only at the study endpoint using a single frequency (50 kHz), these findings should be interpreted cautiously. Future longitudinal studies incorporating baseline and multifrequency EIM analyses will be necessary to better characterize diet-induced alterations in muscle composition and neuromuscular health. Thus, while HFHSD exposure altered extracellular and adipose-related electrical properties, it did not produce clear evidence of overt structural muscle deterioration. Collectively, these findings suggest that the observed reduction in MUNE may reflect early functional impairments in motor neuron–muscle connectivity rather than irreversible motor neuron degeneration.

### 4.3. Potential Physiological Mechanisms Underlying Motor Unit Number Estimation Decline

The following physiological mechanisms are discussed as potential interpretations supported by prior literature, as these pathways were not directly assessed in the present study. Aging-related reductions in muscle strength are known to be selective for type IIb and IIx fibers, and while MUNE methods do not differentiate between fiber types [43], the motor unit remains the fundamental functional unit of neuromuscular integrity and sarcopenia [67]. The reduction in MUNE observed here is consistent with prior evidence of motor unit loss in elderly rodents, including impairments in muscle contractility, neuromuscular junction signaling, and muscle size [13,68]. The approximate 18.9% decline in MUNE over only 10 weeks (range 10.4–26.6%) is substantial, comparable to reductions seen in models of motor neuron disease or advanced neuromuscular aging [16,39]. Adiposity-related filtering effects may contribute to underestimation of smaller motor units, reducing CMAP amplitude and influencing single motor unit potential amplitude [69,70], though this does not fully account for the magnitude of decline. Previous studies have suggested that obesity-related alterations in calcium signaling, activated protein kinase activity, and excitation-contraction coupling may contribute to neuromuscular dysfunction [5]. Transitions toward fast fiber phenotypes have been reported in some obesity and aging models [71,72], but metabolic dysfunction can also arise without fiber-type shifts [5]. With aging, reduced type II fiber size and number, increased denervation, and less successful reinnervation [73] further compound neuromuscular vulnerability.

### 4.4. Muscle-Specific Effects and Variability in Contractile Outcomes

The effects of adiposity on skeletal muscle function are not uniform and depend on muscle location, fiber-type composition, and in vivo mechanical demands. Muscle-specific loading patterns and functional roles influence mechanical performance, potentially explaining the divergent effects observed between forelimb and hindlimb muscles following HFHSD consumption [74,75]. In the present study, HFHSD reduced tetanic contractile torque while paradoxically increasing absolute forelimb grip strength, with no significant effect on hindlimb grip strength (Figure 5). Similar findings have been reported in early or intermediate stages of diet-induced obesity, where absolute strength measures may be preserved or transiently increased despite underlying metabolic and neuromuscular impairment [76,77,78]. This phenomenon is likely driven by increased body mass, greater chronic mechanical loading, and compensatory hypertrophic adaptations rather than true improvements in intrinsic muscle quality or neuromuscular integrity [77,79,80]. Accordingly, the increase in absolute forelimb grip strength should not be interpreted as improved muscle quality, particularly because this effect did not persist after normalization to body mass. However, normalization to total body mass may not fully account for obesity-related alterations in body composition, including differences in lean mass, adiposity, and muscle quality, which were not directly quantified in the present study. Consistent with this interpretation, prior studies indicate that HFHSD induces muscle-specific morphological alterations [81] and that forelimb and hindlimb muscles differ substantially in fiber-type composition and metabolic susceptibility [6,82]. The apparent reduction in absolute forelimb grip strength observed in the control group between weeks 6 and 10 may reflect normal biological variability associated with longitudinal functional testing in aging rodents, particularly given the modest sample size and behavioral sensitivity of grip strength assessments. In addition, variability within the control group at week 10 was relatively small, resulting in minimal visible error bars in Figure 5C. Moreover, behavioral, motivational, and sensory components contribute more prominently to forelimb grip strength assessments [83], potentially rendering this measure more sensitive to compensatory or non-contractile influences. In contrast, hindlimb grip strength may require longer durations of HFHSD exposure to exhibit measurable deterioration, as suggested by prior work in younger rodent models [84].

## 5. Conclusions

This pilot study provides preliminary longitudinal evidence that a high-fat/high-sugar diet (HFHSD), initiated during late middle age, was associated with early neuromuscular decline in female F344 rats prior to the typical onset of age-related motor unit loss. A 10-week HFHSD intervention was associated with reductions in motor unit number estimation, impaired muscle contractility, and altered electrical properties, with several changes already evident after 6 weeks of dietary exposure. Collectively, these findings suggest that obesogenic dietary conditions may increase vulnerability of the aging neuromuscular system and may contribute to early impairments in motor neuron–muscle connectivity before overt structural muscle deterioration becomes apparent. These results support dietary composition as a potential modifiable factor influencing neuromuscular aging trajectories and warrant further mechanistic and translational investigation.

## 6. Limitations and Future Directions

This study has some limitations that should be considered when interpreting the findings. First, although the high-fat/high-sugar diet used in this study (60.3% fat, 18.3% protein, 21.4% carbohydrate) produced clear neuromuscular and adiposity-related effects, individual food intake and caloric consumption per rat were not directly quantified because animals were group-housed (3 rats/cage). In addition, pair-feeding was not implemented; therefore, the present study cannot fully distinguish the effects of diet composition from those of increased energy intake or obesity-related metabolic dysfunction. Metabolic biomarkers such as glucose, insulin, circulating lipids, inflammatory markers, hepatic lipid accumulation, and insulin resistance indices were also not directly measured. Future studies using individual housing, metabolic profiling, and composition-matched control diets will help clarify the specific contribution of dietary composition and caloric intake to neuromuscular dysfunction.

Second, no histological analyses of skeletal muscle or assessments of intramuscular fat were conducted. Similarly, wet muscle mass was not collected at the study endpoint, limiting the ability to determine whether the observed functional impairments were accompanied by changes in muscle size or atrophy. Incorporating muscle fiber morphology, neuromuscular junction structure, inflammatory markers, lipid infiltration, and muscle wet weight measurements would provide greater mechanistic insight into how HFHSD affects muscle quality and motor unit integrity. Future studies integrating muscle wet weight, histological analyses, intramuscular fat quantification, and neuromuscular junction morphology will provide a more comprehensive understanding of HFHSD-induced neuromuscular dysfunction. Likewise, more precise body composition assessments, such as DXA, MRI, or EchoMRI, would allow detailed quantification of fat mass, lean mass, and ectopic fat distribution beyond anthropometric measures. Increased adiposity may also influence surface electrophysiological recordings and potentially contribute to signal attenuation during MUNE assessments.

Another important limitation is that only female rats were included. Because diet-induced obesity, hormonal regulation, and neuromuscular aging are influenced by sex, the present findings may not fully generalize to male animals. Future studies incorporating both sexes will be necessary to determine potential sex-specific responses to HFHSD exposure.

Finally, the 10-week intervention captures early diet-induced alterations but may not reflect the full trajectory of long-term HFHSD exposure. Extending the feeding period, incorporating longitudinal neuromuscular assessments, and studying younger and older age groups would help determine whether early declines in MUNE predict progressive neuromuscular degeneration with prolonged dietary exposure. Future work integrating functional tests, histology, electrophysiology, and imaging, including assessments of bone quality, would provide a more comprehensive understanding of the musculoskeletal consequences of HFHSD-induced metabolic dysregulation.

## Figures and Tables

**Table 1:** Effects of a high-fat/high-sugar diet on body composition, neuromuscular electrophysiology, muscle contractility, and muscle strength in middle-aged F344 rats after 10 weeks. Data are presented as mean ± standard deviation (SD) with corresponding *p*-values for group comparisons. Abbreviations: g = grams; cm = centimeters; mm = millimeters; mV = millivolts; µV = microvolts; CMAP = compound muscle action potential; SMUP = single motor unit potential; MUNE = motor unit number estimation; wt = weight; mN·m = millinewton-meters; NL = normalized.

Outcome Variable	Control (Mean ± SD)	HFHSD (Mean ± SD)	*p*-Value
**Body Composition**			
Body Mass (g)	445.6 ± 22.28	553.1 ± 27.66	0.0046
Abdominal Girth (cm)	21.1 ± 1.06	24.6 ± 1.23	0.0001
Chest Girth (cm)	20.4 ± 1.02	23.4 ± 1.17	0.0002
Scapula Skinfold (mm)	3.53 ± 0.18	4.47 ± 0.22	0.0008
Abdominal Skinfold (mm)	2.2 ± 0.11	2.5 ± 0.12	0.0060
**Neuromuscular Electrophysiology**			
CMAP (mV)	61.8 ± 5.94	56.8 ± 9.09	0.0219
SMUP (µV)	144.2 ± 14.9	168.5 ± 31.1	0.6898
MUNE Hindlimb (# motor units)	370.0 ± 18.5	300.1 ± 15.01	0.0071
**Muscle Contractility**			
Tetanic Torque (mN·m)	240.5 ± 12.03	196.6 ± 9.83	0.0023
NL Tetanic Torque (mN·m/wt)	0.541 ± 0.03	0.392 ± 0.02	0.0005
**Muscle Strength**			
Forelimb Grip (g)	571.1 ± 28.56	716.3 ± 35.81	0.0273
NL Forelimb Grip (g/wt)	1.145 ± 0.06	1.659 ± 0.08	0.1924
Hindlimb Grip Strength (g)	516.6 ± 25.83	579.2 ± 28.96	0.6281
NL Hindlimb Grip Strength (g/wt)	1.174 ± 0.06	1.086 ± 0.05	0.2323

## Data Availability

The original contributions presented in this study are included in the article. Further inquiries can be directed to the corresponding author.

## References

[1] Oike H., Ogawa Y., Azami K. (2020). Long-Term Feeding of a High-Fat Diet Ameliorated Age-Related Phenotypes in SAMP8 Mice. Nutrients.

[2] Moreno-Fernández S., Garcés-Rimón M., Vera G., Astier J., Landrier J.F., Miguel M. (2018). High Fat/High Glucose Diet Induces Metabolic Syndrome in an Experimental Rat Model. Nutrients.

[3] Longo M., Zatterale F., Naderi J., Parrillo L., Formisano P., Raciti G.A., Beguinot F., Miele C. (2019). Adipose Tissue Dysfunction as Determinant of Obesity-Associated Metabolic Complications. Int. J. Mol. Sci..

[4] Pataky M.W., Wang H., Yu C.S., Arias E.B., Ploutz-Snyder R.J., Zheng X., Cartee G.D. (2017). High-Fat Diet-Induced Insulin Resistance in Single Skeletal Muscle Fibers is Fiber Type Selective. Sci. Rep..

[5] Tallis J., James R.S., Seebacher F. (2018). The effects of obesity on skeletal muscle contractile function. J. Exp. Biol..

[6] Messa G.A.M., Piasecki M., Hurst J., Hill C., Tallis J., Degens H. (2020). The impact of a high-fat diet in mice is dependent on duration and age, and differs between muscles. J. Exp. Biol..

[7] McCormick R., Vasilaki A. (2018). Age-related changes in skeletal muscle: Changes to life-style as a therapy. Biogerontology.

[8] Skelton D.A. (2002). Explosive power and asymmetry in leg muscle function in frequent fallers and non-fallers aged over 65. Age Ageing.

[9] Grosicki G.J., Zepeda C.S., Sundberg C.W. (2022). Single muscle fibre contractile function with ageing. J. Physiol..

[10] Cuoco A., Callahan D.M., Sayers S., Frontera W.R., Bean J., Fielding R.A. (2004). Impact of Muscle Power and Force on Gait Speed in Disabled Older Men and Women. J. Gerontol. A Biol. Sci. Med. Sci..

[11] Henderson R.D., McCombe P.A. (2017). Assessment of Motor Units in Neuromuscular Disease. Neurotherapeutics.

[12] Lee W.S., Cheung W.H., Qin L., Tang N., Leung K.S. (2006). Age-associated Decrease of Type IIA/B Human Skeletal Muscle Fibers. Clin. Orthop. Relat. Res..

[13] Bunn J.A. (2012). Aging and the Motor Unit. J. Sports Med. Doping Stud..

[14] Padilla C.J., Harrigan M.E., Harris H., Schwab J.M., Rutkove S.B., Rich M.M., Clark B.C., Arnold W.D. (2021). Profiling age-related muscle weakness and wasting: Neuromuscular junction transmission as a driver of age-related physical decline. Geroscience.

[15] Hotamisligil G.S. (2017). Inflammation, metaflammation and immunometabolic disorders. Nature.

[16] Addison O., Marcus R.L., LaStayo P.C., Ryan A.S. (2014). Intermuscular Fat: A Review of the Consequences and Causes. Int. J. Endocrinol..

[17] Piasecki M., Ireland A., Stashuk D., Hamilton-Wright A., Jones D.A., McPhee J.S. (2016). Age-related neuromuscular changes affecting human vastus lateralis. J. Physiol..

[18] Manini T.M., Clark B.C. (2012). Dynapenia and Aging: An Update. J. Gerontol. Ser. A.

[19] Sertié R.A.L., Caminhotto R de O., Andreotti S., Campaña A.B., de Proença A.R.G., de Castro N.C., Lima F.B. (2015). Metabolic adaptations in the adipose tissue that underlie the body fat mass gain in middle-aged rats. Age.

[20] Chugh D., Iyer C.C., Wang X., Bobbili P., Rich M.M., Arnold W.D. (2020). Neuromuscular junction transmission failure is a late phenotype in aging mice. Neurobiol. Aging.

[21] Fogarty M.J., Porras M.A.G., Mantilla C.B., Sieck G.C. (2019). Diaphragm neuromuscular transmission failure in aged rats. J. Neurophysiol..

[22] Carter L.G., Qi N.R., De Cabo R., Pearson K.J. (2013). Maternal Exercise Improves Insulin Sensitivity in Mature Rat Offspring. Med. Sci. Sports Exerc..

[23] Datta P., Zhang Y., Parousis A., Sharma A., Rossomacha E., Endisha H., Wu B., Kacprzak I., Mahomed N.N., Gandhi R. (2017). High-fat diet-induced acceleration of osteoarthritis is associated with a distinct and sustained plasma metabolite signature. Sci. Rep..

[24] Padilla C.J., Harris H., Volek J.S., Clark B.C., Arnold W.D. (2024). Effects of a ketogenic diet on motor function and motor unit number estimation in aged C57BL/6 mice. J. Nutr. Health Aging.

[25] Sliwowska J.H., Barker J.M., Barha C.K., Lan N., Weinberg J., Galea L.A.M. (2010). Stress-induced suppression of hippocampal neurogenesis in adult male rats is altered by prenatal ethanol exposure. Stress..

[26] Shi Y., Lu Z., Song W., Wang Y., Zhou Q., Geng P., Zhou Y., Wang S., Han A. (2024). The Impact of Baohuoside I on the Metabolism of Tofacitinib in Rats. Drug Des. Devel Ther..

[27] Morgan C.J. (2017). Use of proper statistical techniques for research studies with small samples. Am. J. Physiol.-Lung Cell. Mol. Physiol..

[28] Priego T., Sánchez J., Picó C., Palou A. (2008). Sex-differential Expression of Metabolism-related Genes in Response to a High-fat Diet. Obesity.

[29] Eckel L.A., Moore S.R. (2004). Diet-induced hyperphagia in the rat is influenced by sex and exercise. Am. J. Physiol.-Regul. Integr. Comp. Physiol..

[30] Taraschenko O.D., Maisonneuve I.M., Glick S.D. (2011). Resistance of male Sprague–Dawley rats to sucrose-induced obesity: Effects of 18-methoxycoronaridine. Physiol. Behav..

[31] Roca P., Rodriguez A.M., Oliver P., Bonet M., Quevedo S., Picó C., Palou A. (1999). Brown adipose tissue response to cafeteria diet-feeding involves induction of the UCP2 gene and is impaired in female rats as compared to males. Pflug. Arch. Eur. J. Physiol..

[32] Quirós Cognuck S., Reis W.L., Silva M., Debarba L.K., Mecawi A.S., de Paula F.J., Franci C.R., Elias L.L., Antunes-Rodrigues J. (2020). Sex differences in body composition, metabolism-related hormones, and energy homeostasis during aging in Wistar rats. Physiol. Rep..

[33] Pellizzon M.A., Ricci M.R. (2020). Choice of laboratory rodent diet may confound data interpretation and reproducibility. Curr. Dev. Nutr..

[34] Bastías-Pérez M., Serra D., Herrero L. (2020). Dietary Options for Rodents in the Study of Obesity. Nutrients.

[35] Park S.B., Yang S.J. (2024). Ketogenic diet preserves muscle mass and strength in a mouse model of type 2 diabetes. PLoS ONE.

[36] Tekus E., Miko A., Furedi N., Rostas I., Tenk J., Kiss T., Szitter I., Balasko M., Helyes Z., Wilhelm M. (2018). Body fat of rats of different age groups and nutritional states: Assessment by micro-CT and skinfold thickness. J. Appl. Physiol..

[37] Marshall M.W., Smith B.P., Munson A.W., Lahmanhn R.P. (1969). Prediction of carcass fat from body measurements made on live rats differing in age, sex and strain. Br. J. Nutr..

[38] Owendoff G., Ray A., Bobbili P., Clark L., Baumann C.W., Clark B.C., Arnold W.D. (2023). Optimization and construct validity of approaches to preclinical grip strength testing. J. Cachexia Sarcopenia Muscle.

[39] Sheth K.A., Iyer C.C., Wier C.G., Crum A.E., Bratasz A., Kolb S.J., Clark B.C., Burghes A.H., Arnold W.D. (2018). Muscle strength and size are associated with motor unit connectivity in aged mice. Neurobiol. Aging.

[40] Iyer S.R., Shah S.B., Lovering R.M. (2021). The Neuromuscular Junction: Roles in Aging and Neuromuscular Disease. Int. J. Mol. Sci..

[41] Wier C.G., Crum A.E., Reynolds A.B., Iyer C.C., Chugh D., Palettas M.S., Heilman P.L., Kline D.M., Arnold W.D., Kolb S.J. (2019). Muscle contractility dysfunction precedes loss of motor unit connectivity in SOD1(G93A) mice. Muscle Nerve.

[42] Arnold W.D., Sheth K.A., Wier C.G., Kissel J.T., Burghes A.H., Kolb S.J. (2015). Electrophysiological Motor Unit Number Estimation (MUNE) Measuring Compound Muscle Action Potential (CMAP) in Mouse Hindlimb Muscles. J. Vis. Exp..

[43] Ibrahem A.K., Al-Mahdawi A.M., Hamdan F.B. (2021). Motor unit number estimation versus compound muscle action potential in the evaluation of motor unit loss in amyotrophic lateral sclerosis. Egypt J. Neurol. Psychiatr. Neurosurg..

[44] de Carvalho M., Barkhaus P.E., Nandedkar S.D., Swash M. (2018). Motor unit number estimation (MUNE): Where are we now?. Clin. Neurophysiol..

[45] Gooch C.L., Doherty T.J., Chan K.M., Bromberg M.B., Lewis R.A., Stashuk D.W., Berger M.J., Andary M.T., Daube J.R. (2014). Motor unit number estimation: A technology and literature review. Muscle Nerve.

[46] Hill C., James R.S., Cox V.M., Seebacher F., Tallis J. (2020). Age-related changes in isolated mouse skeletal muscle function are dependent on sex, muscle, and contractility mode. Am. J. Physiol. Regul. Integr. Comp. Physiol..

[47] Ahad M.A., Rutkove S.B. (2009). Electrical impedance myography at 50 kHz in the rat: Technique, reproducibility, and the effects of sciatic injury and recovery. Clin. Neurophysiol..

[48] Sanchez B., Rutkove S.B. (2017). Electrical Impedance Myography and Its Applications in Neuromuscular Disorders. Neurotherapeutics.

[49] Rutkove S.B. (2009). Electrical impedance myography: Background, current state, and future directions. Muscle Nerve.

[50] He M., Wang J., Wang Y., Sui J., Zhang M., Ding X., Zhao Y., Chen Z., Ren X., Shi B. (2020). High-fat diet-induced adipose tissue expansion occurs prior to insulin resistance in C57BL/6J mice. Chronic Dis. Transl. Med..

[51] Kappe C., Zhang Q., Nyström T., Sjöholm Å. (2014). Effects of high-fat diet and the anti-diabetic drug metformin on circulating GLP-1 and the relative number of intestinal L-cells. Diabetol. Metab. Syndr..

[52] Motil K.J., Geerts S., Annese F., Neul J.L., Benke T., Marsh E., Lieberman D., Skinner S.A., Glaze D.G., Heydemann P. (2022). Anthropometric Measures Correspond with Functional Motor Outcomes in Females with Rett Syndrome. J. Pediatr..

[53] Melo B.P., Zacarias A.C., Oliveira J.C.C., de Souza L.M.C., Sabino J., Ferreira A.V.M., Tonoli C., dos Santos M.L., de Avelar G.F., Meeusen R. (2021). Thirty days of combined consumption of a high-fat diet and fructose-rich beverages promotes insulin resistance and modulates inflammatory response and histomorphometry parameters of liver, pancreas, and adipose tissue in Wistar rats. Nutrition.

[54] Han J., Nepal P., Odelade A., Freely F.D., Belton D.M., Graves J.L., Maldonado-Devincci A.M. (2021). High-Fat Diet-Induced Weight Gain, Behavioral Deficits, and Dopamine Changes in Young C57BL/6J Mice. Front Nutr..

[55] Stott N.L., Marino J.S. (2020). High Fat Rodent Models of Type 2 Diabetes: From Rodent to Human. Nutrients.

[56] Sampey B.P., Vanhoose A.M., Winfield H.M., Freemerman A.J., Muehlbauer M.J., Fueger P.T., Newgard C.B., Makowski L. (2011). Cafeteria Diet Is a Robust Model of Human Metabolic Syndrome With Liver and Adipose Inflammation: Comparison to High-Fat Diet. Obesity.

[57] Poggiogalle E., Rossignon F., Carayon A., Capel F., Rigaudière J.-P., Vincent S.D.S., Le-Bacquer O., Salles J., Giraudet C., Patrac V. (2022). Deleterious Effect of High-Fat Diet on Skeletal Muscle Performance Is Prevented by High-Protein Intake in Adult Rats but Not in Old Rats. Front Physiol..

[58] Shi L., Tu B.P. (2015). Acetyl-CoA and the regulation of metabolism: Mechanisms and consequences. Curr. Opin. Cell Biol..

[59] Reis-Costa A., Belew G.D., Viegas I., Tavares L.C., Meneses M.J., Patrício B., Gastaldelli A., Macedo M.P., Jones J.G. (2024). The Effects of Long-Term High Fat and/or High Sugar Feeding on Sources of Postprandial Hepatic Glycogen and Triglyceride Synthesis in Mice. Nutrients.

[60] González-Muniesa P., Milagro F.I., Campión J., Martínez J.A. (2006). Reduction in energy efficiency induced by expression of the uncoupling protein, UCP1, in mouse liver mitochondria. Int. J. Mol. Med..

[61] Kennedy A.R., Pissios P., Otu H., Roberson R., Xue B., Asakura K., Furukawa N., Marino F.E., Liu F.-F., Kahn B.B. (2007). A high-fat, ketogenic diet induces a unique metabolic state in mice. Am. J. Physiol.-Endocrinol. Metab..

[62] Silvennoinen M., Rinnankoski-Tuikka R., Vuento M., Hulmi J.J., Torvinen S., Lehti M., Kivelä R., Kainulainen H. (2013). High-fat feeding induces angiogenesis in skeletal muscle and activates angiogenic pathways in capillaries. Angiogenesis.

[63] Khan I.M., Perrard X.Y., Brunner G., Lui H., Sparks L.M., Smith S.R., Wang X., Shi Z.-Z., Lewis D.E., Wu H. (2015). Intermuscular and perimuscular fat expansion in obesity correlates with skeletal muscle T cell and macrophage infiltration and insulin resistance. Int. J. Obes..

[64] Wallace M.A., Aguirre N.W., Marcotte G.R., Marshall A.G., Baehr L.M., Hughes D.C., Hamilton K.L., Roberts M.N., Lopez-Dominguez J.A., Miller B.F. (2021). The ketogenic diet preserves skeletal muscle with aging in mice. Aging Cell..

[65] Busutil R., Espallardo O., Torres A., Martínez-Galdeano L., Zozaya N., Hidalgo-Vega Á. (2017). The impact of obesity on health-related quality of life in Spain. Health Qual. Life Outcomes.

[66] Clark B.C., Rutkove S., Lupton E.C., Padilla C.J., Arnold W.D. (2021). Potential Utility of Electrical Impedance Myography in Evaluating Age-Related Skeletal Muscle Function Deficits. Front. Physiol..

[67] Fogarty M.J., Marin Mathieu N., Mantilla C.B., Sieck G.C. (2020). Aging reduces succinate dehydrogenase activity in rat type IIx/IIb diaphragm muscle fibers. J. Appl. Physiol..

[68] Baek K.W., Jung Y.K., Kim J.S., Hah Y.-S., Kim S.-J., Yoo J.-I. (2020). Rodent Model of Muscular Atrophy for Sarcopenia Study. J. Bone Metab..

[69] Doherty T.J., Vandervoort A.A., Brown W.F. (1993). Effects of Ageing on the Motor Unit: A Brief Review. Can. J. Appl. Physiol..

[70] Narici M.V., Maffulli N. (2010). Sarcopenia: Characteristics, mechanisms and functional significance. Br. Med. Bull..

[71] Kent-Braun J.A., Ng A.V. (2000). Skeletal muscle oxidative capacity in young and older women and men. J. Appl. Physiol..

[72] Seebacher F., Tallis J., McShea K., James R.S. (2017). Obesity-induced decreases in muscle performance are not reversed by weight loss. Int. J. Obes..

[73] Lee K.Y., Singh M.K., Ussar S., Wetzel P., Hirshman M.F., Goodyear L.J., Kispert A., Kahn C.R. (2015). Tbx15 controls skeletal muscle fibre-type determination and muscle metabolism. Nat. Commun..

[74] Lang T., Streeper T., Cawthon P., Baldwin K., Taaffe D.R., Harris T.B. (2010). Sarcopenia: Etiology, clinical consequences, intervention, and assessment. Osteoporos. Int..

[75] Bollinger L.M. (2017). Potential contributions of skeletal muscle contractile dysfunction to altered biomechanics in obesity. Gait Posture.

[76] Tomlinson D.J., Erskine R.M., Morse C.I., Winwood K., Onambélé-Pearson G. (2016). The impact of obesity on skeletal muscle strength and structure through adolescence to old age. Biogerontology.

[77] Rolland Y., Lauwers-Cances V., Cristini C., van Kan G.A., Janssen I., Morley J.E., Vellas B. (2009). Difficulties with physical function associated with obesity, sarcopenia, and sarcopenic-obesity in community-dwelling elderly women: The EPIDOS (EPIDemiologie de l’OSteoporose) Study. Am. J. Clin. Nutr..

[78] Pua Y.H., Tay L., Clark R.A., Thumboo J., Tay E.-L., Mah S.-M., Ng Y.-S. (2023). Associations of height, weight, and body mass index with handgrip strength: A Bayesian comparison in older adults. Clin. Nutr. ESPEN.

[79] Li Y., Guo W., Li H., Wang Y., Liu X., Kong W. (2025). The Change of Skeletal Muscle Caused by Inflammation in Obesity as the Key Path to Fibrosis: Thoughts on Mechanisms and Intervention Strategies. Biomolecules.

[80] Blaauw B., Schiaffino S., Reggiani C. (2013). Mechanisms modulating skeletal muscle phenotype. Compr. Physiol..

[81] Zuurbier C.J., Huijing P.A. (1993). Changes in geometry of activily shortening unipennate rat gastrocnemius muscle. J. Morphol..

[82] Armstrong R.B., Phelps R.O. (1984). Muscle fiber type composition of the rat hindlimb. Am. J. Anat..

[83] Tam C.S., Power J.E., Markovic T.P., Yee C., Morsch M., McLennan S.V., Twigg S.M. (2015). The effects of high-fat feeding on physical function and skeletal muscle extracellular matrix. Nutr. Diabetes.

[84] Fujiwara M., Yoshito N., Iwata M., Lee-Hotta S., Inoue T., Aizawa Y., Kametaka S., Asai Y., Suzuki S. (2020). Median nerve injury does not contribute to early onset of decreased grip strength due to repetitive reaching and grasping tasks in rats. Neuro Endocrinol. Lett..

